# Prophylaxis of Intestinal Stasis

**Published:** 1914-04

**Authors:** 


Prophylaxis of Intestinal Stasis.—Seton Pringle, the well known Dublin surgeon, is an experienced laparotomist and familiar with the different procedures undertaken to relieve intestinal stasis. All the more weight, therefore, must be given to his advice to prevent the condition, if possible, and to try medicinal agents before employing any of the operative procedures, "legion," as he says, "in number." In the *Dublin Journal of Medical Science* for February, Mr. Pringle impresses upon the profession his belief that the abdominal are the most important voluntary muscles of the body, and if they are properly developed by suitable exercises the possibility of ptosis is reduced to a minimum; if there is any costiveness it should be combated early by the regulation of the diet and the use of liquid paraffin (mineral oil). Many patients, if seen early, may be rendered comfortable by the use of an abdominal belt in addition to the exercise and oil. The latter he gives in doses of one to four drams three times a day, flavored with cinnamon or peppermint, or else advises one of the proprietary preparations.—Exchange.

